# Exploring Patient Organization Involvement in Stem Cell Research: Insights from the Lifeworld

**DOI:** 10.1007/s11673-025-10516-6

**Published:** 2026-03-17

**Authors:** Edilene Lopes, Rachel Ankeny, Dan Santos, Mengqi Hu, Tess Whitton, Michail Ivanov, Joan Leach, Dianne Nicol, Christine Wells

**Affiliations:** 1 School of Humanities, Adelaide University, Napier Building, Level 7, North Terrace, Adelaide, SA 5005 Australia; 2 Wageningen University, Hollandseweg 1(Building 201), 6706 KN Wageningen, The Netherlands; 3 Australian National Centre for the Public Awareness of Science, Australian National University, 42a Linnaeus Way, Acton, ACT 2601 Australia; 4 Stem Cell Systems, Anatomy and Physiology, Faculty of Medicine Dentistry and Health Sciences, University of Melbourne, Room 2.11, Level 2, Kenneth Myer Building, Parkville, VIC 3010 Australia; 5 Melbourne Law School, University of Melbourne, 185 Pelham Street, Carlton, VIC 3053 Australia; 6 Faculty of Law, University of Tasmania, Private Bag 89, Hobart, TAS 7001 Australia

**Keywords:** Stem cell, Patient organizations, Patient involvement, Bioethics, Habermas, Australia

## Abstract

This article examines the role of patient organizations as mediators between patients and researchers in stem cell research in Australia. We conducted semi-structured interviews with stem cell researchers and patient organization representatives as part of a broader project to deepen public understanding and foster more robust scientific practices in stem cell-based research and therapies. We used Habermas’s Theory of Communicative Action as our theoretical framework to explore the relationships between patient organizations and researchers. Using this framework allowed the identification of two different ways of conceptualizing health and illness—one based on patients’ experiences, which we call “the lifeworld,” and another grounded on researchers’, health professionals’, and policymakers’ professional perspectives, which we call “the rational system.” We found that the different conceptualizations identified influenced the interactions between patients and researchers (and other professionals involved in the healthcare system). Patient organizations, then, act as articulators of patients’ viewpoints in this scenario, which can have advantages and disadvantages. We also found that researchers need to develop less transactional forms of communication with these organizations and that policymakers and regulators could benefit from developing guidelines to encourage researchers to use diverse strategies to engage more meaningfully with patients at individual and organizational levels.

**Supplementary Information:**

The online version contains supplementary material available at https://doi.org/10.1007/s11673-025-10516-6.

## Introduction

Induced Pluripotent Stem Cells (iPSCs) are increasingly being used in clinical research projects involving human participants. iPSCs have been hailed by some as promising alternatives that also avoid many traditional ethical concerns associated with research involving embryonic stem cells (Devolder 2009). However, some studies have pointed to other ethical issues, such as ensuring participant privacy, obtaining informed consent for such complex research, and potential commercialization of research outputs (Moradi et al. 2019). These concerns have resulted in discussions about how to increase public and patient involvement in this type of research and its governance (Dasgupta et al. 2014). In many instances, patient organizations help to establish links between patients and researchers (Baggott and Forster 2008), but their role in these processes has not yet been explored in the scholarly literature on stem cell research.

Patient organizations typically sit between the embodied subjective experiences of patients and societal requirements associated with ensuring that health and medical research is overseen and performed ethically and equitably (Brown et al. 2004). In this paper, we examine the role of patient organization representatives as mediators between individual patients’ experiences and researchers’ views, using stem cell research as our focus.^1^ There is a broad literature assessing the roles of patient organizations in the healthcare system and research: some see them as helping to improve patient health outcomes, but others view their status as somewhat more problematic, as they do not necessarily represent patient interests alone due to their hybrid motivations (Baggott and Forster 2008; Pinto et al. 2016). This paper argues that these patterns and tensions can be usefully contextualized and better understood using Habermas’s Theory of Communicative Action (1984, 1987). We contend that many concerns about patient organizations arise because their staff or volunteers convey patients’ perspectives about health and illness which are often fundamentally different from the views of those individuals working in healthcare or medical research (see also Namazi et al. 2016; van Dulmen 2002), as reflected in Habermas’s distinction between “the lifeworld” and “rational systems.” This framework enables a deeper understanding of the interactions between patient organization representatives and stem cell researchers, which are grounded in different rationales and are also influenced by specific contexts (e.g., organizational rules that individuals need to follow). These broader factors partially explain their mismatched expectations and the resulting tensions we can observe. However, engagement between researchers and patient representatives takes place in a shared space where, as individuals, they forge collaborations in an attempt to achieve their respective objectives. We argue that recognizing this dynamic can provide opportunities for developing strategies to address some of these identified challenges.

## Background

Patient and public involvement (PPI) in health-related research is not a new trend, as it dates back to the late 1960 s (Kelleher 2001). However, there are still no established standards for best practices in engagement methods or how to measure the outcomes and impacts of PPI on research. Since the early 2000 s, patient involvement in healthcare has been officially recognized as an objective in the Australian Charter of Health Care Rights (Delaney 2018). Recent research findings have shown (perhaps unsurprisingly) that patient engagement improves the quality of research, including the development of user-relevant research objectives, more appropriate recruitment strategies, and more effective implementation of study results (Brett et al. 2014). These findings have led funding agencies to require that researchers increase patient involvement in their projects (Hamilton et al. 2018). Nevertheless, studies on patient involvement, including in medical and health research in Australia where our research was performed, have demonstrated the need for more patient engagement both in research and healthcare settings (Miller et al. 2017; Wiles et al. 2022).

One way in which researchers have been increasing patient participation in both basic and clinical research planning and governance is by developing relationships with patient organizations (Rach et al. 2020). Patient organizations originally developed in the 1940 s in North America and Western Europe as self-help groups based on the Alcoholics Anonymous model of peer support (Rabeharisoa 2008). In the 1960 s, in alignment with other nascent social movements, health self-help groups evolved from a focus on individuals to the development of collective identities, with patients self-identifying as part of a group with specific characteristics and needs. This development, in turn, enabled them to take part in public debates (Rabeharisoa 2008) and resulted in a movement called “health activism,” in which organized groups of patients advocate for the inclusion of patient-centred views alongside the perspectives of governments or health experts in health-related decision-making (Vicari and Cappai 2016). In a more recent trend, patient groups have sought to be recognized as relevant experts about their own conditions by various types of authorities, including government representatives, researchers, and medical and allied health professionals (Rabeharisoa 2008).

Some authors have argued that patient organization personnel are well placed to act as intermediaries between researchers and individual patients (Ayton et al. 2022; Boote et al. 2002). van de Bovenkamp and Vollaard (2018) argue that patient organizations are best placed to represent patients’ perspectives in some circumstances. However, criticisms have also been noted. For instance, Rojatz and Forster (2017) define self-help organizations as advocacy groups that are member-governed, indication-specific (i.e., focused on a specific or a few closely related conditions), and voluntary, concluding that only those organizations that fulfil these criteria are legitimate patient representatives. However, this definition excludes organizations with more formalized structures, for instance those which hire professionals as staff members. Maguire and Britten (2017) see the issue of representation of patients in healthcare domains as contested because there are many diverse understandings of what counts as “representation” and because it is typically impossible to fulfil various idealized criteria for representation. Despite these difficulties, they do note that individuals involved with these organizations can make patient perspectives visible in health research processes and hence can be valuable.

The relationships between patient organizations and other parties also have come under considerable scrutiny. In their exploration of U.S. patient advocacy groups and their financial ties with drug, device, and biotechnology companies, McCoy and colleagues (2017) conclude that there is a great risk of conflicts of interest due to the large amount of financial support that these groups receive and the lack of transparency for reporting such information. In Australia, Fabbri and collaborators (2019) found that pharmaceutical and health-related private companies frequently provided sponsorship to patient organizations; as they note, Australia does have some mechanisms to support transparency practices surrounding these types of funding arrangements, although largely via industry self-regulation.

Despite these concerns, the involvement of patient organizations in health research and policy is widespread in Australia and worldwide (Baggott and Forster 2008; Martin and Tipton 2007; Pinto et al. 2016). The move toward including patients in healthcare and research can also be understood as related to changes in views of health research governance, which was traditionally performed by authorities or experts. In many contexts, these structures have been replaced by other governance models to address the so-called “democratic deficit” by involving affected citizens more directly in decision-making processes (Barbazza and Tello 2014), a model which has support from the World Health Organization (Pang et al. 2003; World Health Organization 1978). In Australia, individuals representing patient organizations are routinely involved in research processes, particularly as advisors or co-investigators, due in no small part to requirements imposed by Australian governmental funding bodies (MRFF 2023; NHMRC 2018). Thus, they are important social actors in the health research governance space, particularly in Australia.

## Theoretical Approach

**Fig. 1:**
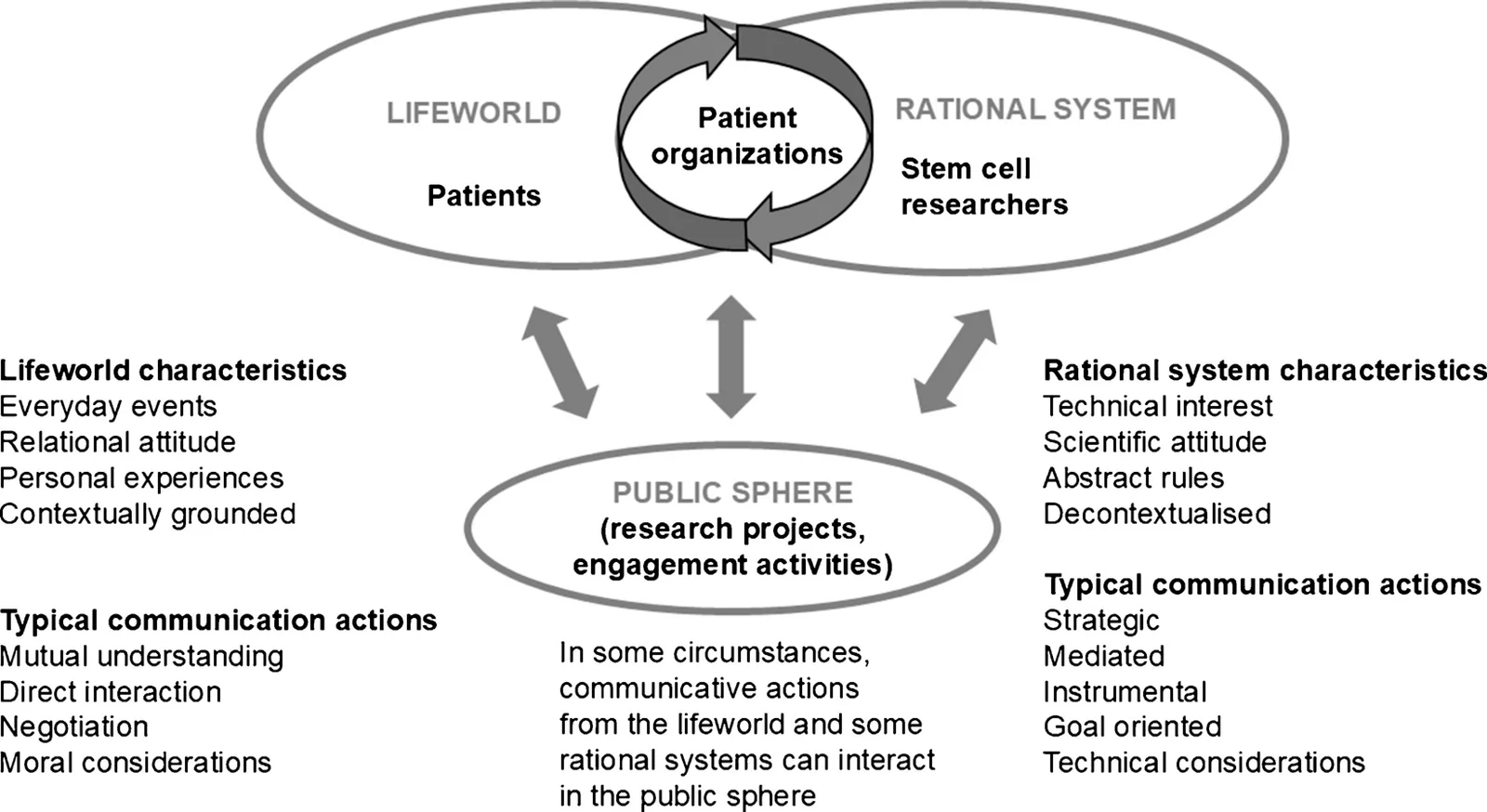
Habermasian concepts used in this article (Habermas 1984, 1987)

In this paper, we use Habermas’s Theory of Communicative Action (1984, 1987) to provide an analytical frame for our empirical findings and their analysis (see Fig. 1). This theory holds that people take part in communicative actions in everyday situations to reach mutual understandings that enable day-to-day activities to occur. These actions are not exclusively individualistic or subjectivistic, nor merely objective and instrumental. Habermas employs the term “lifeworld” to describe the space in which individuals’ external (objective) and internal (subjective) worlds intertwine and interact with other individuals’ worlds:

The lifeworld is, so to speak, the transcendental site where speaker and hearer meet, where they can reciprocally raise claims that their utterances fit the world (objective, social, or subjective), and where they can criticize and confirm those validity claims, settle their disagreements, and arrive at agreements. (Habermas 1987, 126)

The lifeworld’s general principles are embedded in the everyday experiences through which people interact directly with one another. In complex societies, direct interactions happen at the primary level of socialization, but indirect interactions are also necessary for the functioning of society. Such indirect interactions are linked to what Habermas calls “rational systems,” which provide abstract rules that support the indirect interactions necessary for a complex society. For instance, the educational system teaches people the formal skills that are necessary for the functioning of society as well as acceptable ways of socializing outside of the family system. The rules of these systems are not bound by one-on-one mutual understandings (as is the case in the lifeworld) but by rational rules that are learned and accepted by those who take part, which means that these rational systems are structured to meet specific societal objectives. So, in the example of the educational system, the overall objective is for people to learn specific skills that are useful as well the accepted ways of interacting with others in more formal settings which enable a well-functioning society.

The lifeworld and various rational systems co-exist as different but interconnected domains of social life. Individuals are always part of the lifeworld system and perform these types of communicative actions in their everyday direct interactions in local social circles (e.g., with family or friends or in other specific types of social groupings). In other arenas, such as in their professional roles or when interacting with other rational systems, they usually employ rational system communicative actions (Habermas 1987). The correct uses of both types of communicative actions are reinforced through social norms. While the lifeworld remains connected to concrete, mutual understandings through communication, the rational system includes forms of communication that are usually mediated by other mechanisms (such as monetary transactions or bureaucracies). The communicative action of the lifeworld occurs in the public sphere. However, the public sphere can also be the space where important interactions between the lifeworld and rational systems occur, as according to Habermas: “The public sphere in the political realm evolved … through the vehicle of public opinion, it *put*[s] *the state in touch with the needs of society*” (Habermas 1989, 30–31).

The Theory of Communicative Action has increasingly been used to examine practices and implicit structures in different types of institutions and interactions between groups of individuals, including in medicine and healthcare. For instance, Bonell and Melendez-Torres (2023) use it to examine the rationality embedded in evidence-based medicine practices, which serve as the basis of the healthcare system and medical research organization practices in the modern state. The theory also provides a useful method for recognizing the role of other types of knowledge generated in social interactions that work under different principles. For instance, Scambler and Kelleher (2006) explore how Habermasian concepts can inform what they term “New Social Movements (NSM).” NSMs, including user or self-health groups, act as intermediaries between the rational systems and the lifeworld in the health realm, which help challenge rational norms by offering alternative agendas for action. The Theory of Communicative Action is particularly useful in cases where patients’ lived experiences come into contact—and perhaps exist in tension—with rational systems activities, such as in the case of stem cell research-associated practices presented in this paper, and for explaining how certain types of actors can serve as mediators between these systems.

Patients’ individual experiences of health and illness can be viewed as part of the lifeworld, as Little et al. (2022) have discussed in their work on the discourse of experience. These experiences occur when someone (at an individual level) identifies as a patient or as having a particular health condition but can also occur at a social level, when individuals come together as a group, via self-help groups or patient organizations, to express their individual views and describe their experiences.

In contrast to the individual level of interaction, the provision of services within a public healthcare system aimed at maintaining health or minimizing illness—including doing research in these domains—requires organized and formalized actions by governments or other types of institutions, such as universities or research entities. While at work, individuals who work in these domains tend to perform communicative actions grounded in their professional rules rather than the principles which govern their communicative actions as individuals. Hence, these actions (including those performed via practices, policies, and diverse forms of governance mechanisms) can be seen as part of the rational system.

When viewed through this analytical lens, the position of patient organization representatives presents an interesting case, because they are not solely representing an individual nor are they strictly part of the rational system. We contend that these individuals are best understood as situated in the space between the lifeworld of patients and the rational system of healthcare services and research in the public sphere, as they use strategies that are typical of the lifeworld (e.g., connections with individual researchers) and strategies that are part of rational systems repertoire (e.g., offering grants with contractual rules to researchers), depending on which types of organizations they are connected with (e.g., small/self-help groups or larger/professionally-run institutions).

It is important to note that patient organization representatives act within institutions that are not homogeneous. Some patient groups are organized as peer self-help groups, with a focus on providing safe spaces for people who are “like them” to talk and exchange experiences (e.g., see Landzelius 2006). Some more formal patient organizations act as advocacy groups representing patients in policy and research decision-making domains (Levy 2000) and, therefore, are more closely aligned with the structures and communicative actions associated with rational systems in medicine and healthcare. Hence, the different structures and goals of patient groups are of relevance, as they position their representatives at various points along a spectrum of forms of communication, from individual to institutional, with implications for how their interactions with researchers, representatives of health/medical research organizations, industry, and government take place.

However, despite these differences, we argue that both extremes of the spectrum associated with patient organizations explicitly or implicitly generate resistance to (over)rationalization of the lifeworld by forcing consideration of diverse patient experiences in the context of decisions made within medicine and healthcare provision and research. In other words, patient organization representatives affirm the presence of the lifeworld within the rational system (Scambler and Kelleher 2006). This can also happen when individual researchers and patients interact directly without the intervention of third parties, as these experiences can then also have effects on the rational system through the actions of researchers acting in their professional roles.

Consequently, we contend that a Habermasian lens is a useful device for examining how researchers and patient organization representatives involved in stem cell research describe their interactions and collaborations, including the challenges that result. For example, rapid advances in this field make it difficult to predict impacts in the mid- to longer-term, and this level of uncertainty is viewed as an obstacle to patient involvement. Challenges in recruiting participants to donate biomaterials for research projects are usually attributed to the fact that basic research is far removed from patients’ expectations. Through our empirical analysis using Habermas’ theory, we show that these mismatched expectations between patients and researchers have an underlying cause; namely, the different structures in which they are acting and experiencing life, and the different models of communicative action associated with the lifeworld and rational systems.

## Methods

### Recruitment

Ethics approval for this project was obtained from an Australian human research ethics committee at the University of Adelaide (approval number H-2022–095). We interviewed stem cell researchers across a range of career stages, defining “stem cell researchers” as scientists working with stem cell lines that have been developed by themselves or others (manufacturers), or in clinical settings (clinicians). We identified researchers via publicly available information in stem cell registries, funding agencies’ documents (e.g., Australian Research Council), previously funded national programmes (e.g., the Stem Cells Australia initiative), and relevant events and networks (e.g., the Australasian Society for Stem Cell Research [ASSCR]). Of the sixty-one potential interviewees contacted, forty-eight agreed to participate; reasons for non-participation included lack of response, relocation to outside of Australia, or inappropriateness (and suggestions of alternative interviewees).

Patient organization representatives were recruited through a purposive sampling technique, again using publicly available information. The diseases or disorders mentioned by stem cell researchers in their interviews as targets of research including for stem cell-based therapeutics served as the starting points for identifying organizations. Subsequently, a maximum variation sampling technique (Liamputtong and Ezzy 2005) was adopted to increase the diversity of organizations in terms of geographic location and types (e.g., small versus large organizations), based on Australian Government Fair Work Ombudsman (2022) and Australian Charities and Not-for-profits Commission (2022) definitions. Initially, thirty-five potential participants were approached and seventeen agreed to participate; reasons for non-participation included non-response and scheduling difficulties.

For both groups, recruitment stopped when thematic saturation was reached without a predetermined quota. Table 1 presents the participants’ characteristics:

**Table 1:** Participants’ characteristics

Patient Organizations	Size	Conditions	Location
8	Small (S)	Arthritis, Batten Disease, Cerebral Palsy, Diabetes, Epilepsy, Friedreich Ataxia, Haemophilia, Leukaemia, Liver Conditions, Macular Diseases, Motor Neurone Disease, Multiple Sclerosis, Muscular Dystrophy, Parkinson’s	Sydney, Adelaide, Melbourne, Brisbane, Canberra
7	Large (L)		
Stem Cell Researchers		Role (Including Dual Roles)^a^	Location
48		Researcher (3 also worked as clinicians and 4 also worked as manufacturers of stem cell lines)	Melbourne, Sydney/Wollongong, Brisbane, Hobart, Perth, Adelaide

^a^ Some participants were identified as having more than one role

### Data Collection

The data collection occurred from July 2022 to April 2023 (stem cell researchers) and April 2023 to August 2024 (patient organization representatives). Interviews were conducted face-to-face or electronically via Zoom, were video- or audio-recorded, and transcribed with the use of AI software (otter.ai) followed by manual clean up by a researcher. The interviews with stem cell researchers elicited participants’ perspectives on their interactions with patient organization representatives amongst other topics (e.g., openness and collaboration, commercialization, and use of stem cell registries; see Hu et al. 2024; Santos et al. 2025). Interviews with patient organization representatives included questions related to access to and the provision of health research information, involvement in research (including stem cell-related research in particular), collaboration, ethical concerns, and commercialization.

### Data Analysis

A blended approach of inductive and deductive coding was adopted (Braun and Clarke 2022), with codes reflecting a pre-coded list based on themes from the co-authors’ initial discussions and the underlying project framework as well as emergent ideas from the data. For patient organization representative and researcher interviews, three co-authors (EL, DS and RA) separately coded three interviews, and discussed coding and theme-structures. Interviews with stem cell researchers were then coded by DS and those with patient organization representatives by EL. All transcripts were coded using the initial codes and emergent codes. Each code was revised for consistency and mapped under broader theme-structures to arrive at a final version of the data files. Data were analysed using NVivo software.

## Findings

Our broad findings identified strongly contrasting views expressed by the patient organization representatives and stem cell researchers interviewed, and these can be usefully mapped using Habermas’ Theory of Communicative Action. In particular, we focus on how these two groups of individuals used different types of communicative actions that were more consistent either with the lifeworld or the rational system, and expand on the implications of these differences concerning understanding in communication, collaboration, and ethical considerations.

### Patient Organization Representatives: Navigating the Lifeworld and the Rational System

Patient organization representatives position themselves as intermediaries between individual patients and other parties, including researchers. These individuals place great emphasis on collaborating with various actors and advocating for bringing a “patient perspective” to policy, research, and the development of new health products and services. In this sense, they promote the incorporation of lifeworld concerns (individual, patient-related) into rational systems (e.g., healthcare organizations and governmental research priorities) (Pinto et al. 2016).

In this study, representatives of large and small patient organizations explained that they considered engagement with individual researchers to be an important part of their work for a range of reasons, such as being kept informed of the latest advances or new research directions related to various health conditions. Direct interactions with other individuals are one means through which lifeworld mutual understandings are reached. Thus, patient organization representatives, regardless of their organization’s structure or size, utilize interactions with individuals as part of their repertoire for accessing the broader, rational system: “…there’s ongoing discussion and communication with those researchers internationally and locally. This requires a combination of keeping across the literature, the patient and family community, clinical research developments and the academic research community” (PR25—patient organization representative).

Rational system communicative actions are also utilized by our interviewees, such as providing funding to researchers to influence what topics are pursued and to bring patient concerns into research processes. This type of communicative action is typical in the healthcare and research rational systems, where formal contracts are established via transactional elements, and financial rewards are provided in exchange for technical expertise and promoting priorities. Mention of these strategies were typical of representatives of large patient organizations, which are more versed in transitioning between rational system and lifeworld strategies:

We have a had a research grant programme. (…) Each round, we look at the themes of research that we would like to fund, and they tend to be aligned to the [government disease-specific strategy], as well as the national health strategies, as well as to our own strategy. (…) we do require our researchers to have community representation. (…) So, when we’re ranking and scoring research grants, there’s often two or three in the room when our research grant Advisory Committee is discussing research applications, and what should or shouldn’t be funded, or how it’s funded or pros and cons of the application. There’s a strong community rep voice there. (PR04—patient organization representative)

In contrast, patient organization representatives linked to smaller organizations discussed their attempts to shape research priorities mostly by fostering direct interactions with researchers, a type of communicative action that is more firmly rooted in the lifeworld:

I think that our network in that space is quite strong and engaged. And from there, the researchers in that group are able to say: “Okay, well, this is quite a gap. So, let’s see if we can get some funding to do a research piece on this.” (…) And it might be, you know, publishing a commentary piece on something else. So that’s how we sort of work collaboratively … So being able to, again, have someone on the steering committee in that project to really ensure that consumer voice is represented in that research as well. So yeah, that’s how we’re doing [it]. (PR02—patient organization representative)

### Patient Organization Representatives: Recruitment and Patient Involvement in Research

Self-help group representatives, which are comprised mostly of volunteers and focus their attention on one-on-one relationships with individual patients, tend to use communication strategies that are based on contextualized, local understandings that are the hallmark of lifeworld communication practices. Staff from patient organizations with more formal and hierarchized structures tend to use formal channels of communication that are more akin to the communicative actions characteristic of rational systems (Rojatz and Forster 2017). In our study, differences were also noted between the communicative actions of individuals associated with organizations representing diverse conditions: those representing conditions for which no current treatments are available tend to prioritize basic stem cell research, as it is seen as a means for the future development of therapies. For interviewees associated with organizations representing conditions where alternative treatments and research pathways are available, involvement in stem cell research projects was viewed differently.

Patient organization representatives explained that they are generally contacted by researchers to assist in recruiting patients for research projects. According to interviewees, the ways in which they approach requests for assistance in this area vary. Some individuals prefer not to be directly involved and only publicize research projects so long as they are satisfied that minimal ethics requirements are met, such as having approval by a human research ethics committee. However, most of our interviewees stated that they actively participated in recruitment processes:

We are a member-based organization who [sic] supports about 21,000 people living with a chronic condition. It is very common for researchers to approach us for advice, particularly around promotion of their research and recruitment of people. When approached, we work with researchers to advise them the best channels we can use to promote their research. Where needed, we can target the promotion of research to [the] right population within our member-base. (PR04—patient organization representative)

Patient organization representatives of smaller groups also described how they assist researchers to engage patients in other aspects of the research process, such as participating in advisory boards (direct interaction, which is typical of lifeworld-type of communication action):

We also have a service where we connect researchers with advocates [who] are consumers with the lived experience of [condition], but that’s not for participation, that’s more research involvement. So not to participate, but to be part of research teams or to inform the development of different bits of projects and things like that. (PR20—patient organization representative)

### Patient Organization Representatives: Ethical Concerns

When participating in research, patient organization representatives need to reinterpret the lived experiences of patients to inform the rational system, and reinterpret the rational communicative actions of researchers so as to make them more easily accessible to patients. These individuals actively articulate the ideas expressed in both worlds through the processes needed to build relationships between patients and rational system social actors. These articulations include: details about the social identities constructed around patienthood, the disease or condition in question, and the value placed on different types of research (e.g., basic versus clinically oriented) (Landzelius 2006). We found that these considerations affected the interactions between representatives from diverse types of patient organizations and stem cell researchers. For instance, when considering the ethical implications of conducting research with stem cells, the need to reinterpret the lived experiences of patients was explicitly articulated by the patient organization interviewees. The diversity of views on consent and privacy requirements for patients in relation to stem cell research quickly became apparent in this context:

It’s a really complex point. And I think there’s a couple of camps. And I think rightly so. We take the most prudent view and the most conservative view, when we think about the ethical implications, and that’s appropriate. From a sort of research sector point of view, absolutely, that’s appropriate. I think taking the patient view, there are different groups of people. And so, I don’t think all patients are the same. (PR32—patient organization representative)

### Stem Cell Researchers’ Perspectives: Accommodations Made by Rational System Actors

Our stem cell researcher interviewees presented diverse views about the need for collaboration with representatives of patient organizations. The majority of interviewees saw this type of engagement as a necessary step for conducting their research, even though their reasons differed from those provided by the patient organizations representatives. They also were more aligned with ideas related to the transactional nature of healthcare and the rational systems associated with medical research, such as the processes associated with educating patients, fulfilling funding agencies’ requirements, and recruiting research participants:

You want to conduct research that [has] public support and involves patient engagement. I think [this] has become a key criterion for funding agencies, with patients or public representatives often included in the assessment teams. The goal is to address real medical problems, not just satisfy curiosity. In my recent experience with [grant name], a significant portion of the evaluation focuses on patient communication and empowerment, with a significant amount of the score coming from consumer input. (RS01—stem cell researcher)

And the conversation goes the other way, too, particularly in things like stem cells, is educating consumers through those groups as well. You know, … it’s a pretty new field, right and it’s growing so quickly. And I think people often have misconceptions about what stem cell research means. So having that opportunity to interact with the public through these consumer groups and teach them a bit about the value and potential gain we can have from stem cell research is good. That’s basically a two-way conversation. (RS16—stem cell researcher)

However, at the individual level, some of the lifeworld strategies were employed in interactions between researchers and patient organization representatives. Stem cell researchers interviewed did note that they used their interactions with patient organization staff or volunteers to adopt more reflexive approaches to their own work, demonstrating that their direct connections with patients’ lifeworlds influences their actions in the rational system:

And if you engage with those groups, then you can say on your application, “This concept is something that’s important to patients.” I think that’s important because, at the end of the day, you can get caught up in your own little world too much researching things that you may find that patients aren’t as enthusiastic about. So, it’s important to have that balance. (RS04—stem cell researcher)

### The Resistance of Rational Systems to Lifeworld Strategies

Patient organization interviewees mentioned that maintaining relationships with representatives of pharmaceutical or medical companies allows them to be kept informed of new possibilities for treatments, and can also provide sources of income. This type of interaction can be a challenge for staff and volunteers from patient organizations due to concerns, on the part of representatives of government departments and research funding agencies (realized through rational communicative actions such as formal contracts), about whether they are promoting the concerns of patients (legitimate lifeworld concerns) or have been co-opted by the interests of other social actors (mainly individuals representing the interests of private companies). Collaborating with others to reach a mutual understanding and achieve shared goals is a typical lifeworld strategy. However, such collaborations can be seen as a form of collusion which contravenes the norms of the healthcare rational system, where defined roles for individuals associated with specific types of organizations are paramount for the work of the system. In our study—which involved a mix of patient organizational structures ranging from self-help structures run by volunteers to large organizations run by professional staff—all patient organization representatives used collaboration, in one form or another, to reach their goals. Such interactions can damage their attempts to influence healthcare research and policy if they are not seen as representing genuine patient concerns. To counteract calls of potential or actual conflicts of interest in their actions, patient organization interviewees mentioned strategies they use to maintain their “independence,” particularly from individuals representing the interests of private companies as they are seen as more problematic by the other rational system actors:

So, we are in the [medical condition] space, we’ve got relationships with all of the main companies that have commercial products at the moment. And we do often have current connections and contacts with companies that are exploring this space … For the commercial partners, they are supporting some of our programs and projects. And so, we decide the kind of programs that we would like to do that we feel as a need for our community. And then we’ll speak with the various sponsors, to see if there’s something that they’re willing to work to fund and support us on. But we do have a priority, it’s our independence, we are doing the things that we believe we need to do for the [condition] community and offer the opportunity for some of the companies to sponsor. (PR07—patient organization representative)

The efforts of patient organization representatives to interact with health researchers occur in a space where the dominant form of communicative action is that of the rational system. The way that rational system actors (researchers, policymakers, and representatives from funding agencies and medical/pharmaceutical companies) conceptualize health concerns and potential solutions may conflict, at times, with how patients understand their lived experiences and potential strategies for improving them. Some patient organization representatives perceived their interactions with researchers as one-sided, since they tended to be limited to the beginning of a research project. These participants explained that they want patient contributions to research to be more explicitly valued but that the interactions with researchers often fell short of this goal. To put it in another way, they wanted the lifeworld experiences of patients to be recognized as relevant in the rational system framing of health research presented by stem cell researchers:

… we had one project, for example, where they were wanting consumer input. But they were wanting quite a lot of input without actually giving the client any remuneration, or compensation for their time. Which is another thing that we’re pretty passionate about is actually, you know, not just using, you know, people as a tick box: “Oh, yes, you know, we’ve talked to someone from the community,” and actually, making sure they feel valued. (PR09—patient organization representative)

When discussing how information related to stem cell research or wider knowledge of research processes in this area is presented, many researchers mentioned the need to educate patients and the public about stem cells to facilitate involvement in research and inform them about the current state of therapeutic and clinical applications. Again, this framing highlights the typical communicative actions of the healthcare and research rational systems. Educating others not only to understand but to accept or endorse formal norms is a typical communicative action from the medical/health rational system:

“Why am I giving you my skin biopsy? What is the potential to deliver and why am I not better tomorrow?” So, we have our obligation to—it’s something [name] is very passionate about, to explain the methodologies that we use in a way that can be appreciated and the expectations, the future we’re trying to build for the patient groups we’re trying to treat. That’s important. (RS02—stem cell researcher)

## Discussion

As noted in the introduction and theoretical background sections, the role of patient organization representatives as intermediaries between patients and other social actors has been widely discussed in the healthcare literature. Using a Habermasian theoretical lens enables a richer approach to analysing these processes, particularly the communication patterns utilized by the two groups of participants in our study. Our analysis showed that there are tensions and problems in current interactions between representatives of patient organizations and researchers in the stem cell research space. The structure, size, and self-identity of patient organizations as mediators is important for understanding how their representatives communicate with researchers and how they approach their involvement in medical/healthcare research. However, the diversity and complexity in terms of structures, health conditions represented, and goals means that strategies to engage these individuals must be specific, nuanced, and allow for diverse types of knowledge, including lifeworld experiences, to be included.

This study’s findings also underscore the fact that rational system actors can develop alternative forms of communicative actions and relationships not solely based on rational parameters and make adaptations to allow these other types of inputs to inform the logic of the rational system. For example, researchers should explicitly consider how they interact with patient organization representatives and individual patients. The dominant one-way, transactional mode of communication, where rational concerns are considered to be the predominant issue, does not allow other concerns to inform research, even though some researchers mentioned the potential relevance of these inputs to their own work.

In research with stem cells, there are a few recognized ethical challenges that could benefit from incorporating more patient (lifeworld) inputs. According to Sugarman and colleagues (2023), these include the potential for immortal cell lines and the diverse uses to which lines can be put (Moradi et al. 2019); development of therapeutics for rare conditions, including regenerative and personalized medicine, that, if not done carefully, can increase inequality among diverse population subgroups (Fung and Kerridge 2013; Shi et al. 2017); conflicts associated with the commercialization of research processes and outcomes; and how to ensure alignment between patient and public views (Heyder et al. 2020). In our study, patient organization representatives were able to point to ethical concerns that varied depending on the patient, with some more concerned about privacy issues than others, for example. The input from staff working for organizations with more formalized structures should be regarded as equally valuable as that from individuals volunteering at smaller self-help organizations with a diverse collective identity. Utilizing different forms of engagement initiatives and ensuring that a diversity of voices is heard is critical to meeting goals related to integrating patient perspectives into stem cell research priorities and practices.

The use of this Habermasian approach for our analysis should allow researchers, as well as policymakers and funding agency staff, to reflect on how their practices, regulations, or requirements can foster (or hinder) the development of genuine and long-term relationships between patients, their representatives, and researchers. Openness to different viewpoints on how health issues are considered in a research project (e.g., not viewing matters only through a medical lens but also considering the lived perspectives of patients), including flexibility about taking on proposed ideas and building ongoing dialogues with patients and their representatives that can influence the development of a research project, are strategies that can increase research impacts in the lifeworld. Such approaches demand the use of diverse communicative actions that allow for both lifeworld and rational systems concerns to be voiced, discussed, and addressed.

In the case of stem cell research, discussions about the need to engage patients in research associated with rapidly evolving parts of this field has been a recent topic of interest to the ISSCR, the major professional organization representing stem cell researchers (Sugarman et al. 2023). These discussions originated from ethical dilemmas mentioned above, as standards on how to best respond have not progressed as rapidly as needed given the evolving nature of the research. Most ethical issues are currently addressed solely through individual informed consent processes (which could be viewed as formal contracts, a type of communicative action that largely resides in the rational system rather than in the lifeworld). These processes have clear limitations, as has been demonstrated in biobanking research (Kaye 2012). As our interviewees noted, current informed consent practices are not adequate to address many issues associated with uncertainties related to current or future research with iPSCs. As such, there is a need for more ongoing patient engagement to address them adequately. Processes that are based primarily on the rational system way of working give limited attention to the views of patients rooted in their diverse lived experiences and will tend to incentivize transactional interactions between researchers, representatives of pharmaceutical companies, and patient organizations. Procedures that allow for the ongoing adaptability of research proposals and research outputs throughout the life of a research project can facilitate the inclusion of different individuals and inputs into the research process, including from the lifeworlds of patients.

## Study Limitations

In this study, we utilized semi-structured interviews to explore individuals’ perspectives and understandings regarding their own communicative actions and their views of the communicative actions of others. There are some recognized limitations when using this type of data in theoretical frameworks that address how individuals explain their actions, including the potential for deception and intentionality bias, to name just two (Small and Cook 2021). However, our focus in this project was not on uncovering internal motivations or deliberate decisions that lead to communicative actions. Instead, we aimed to understand the way in which participants expressed understandings, during the interviews, which provide a glimpse of the contextual factors that influence these perceptions. While there are other frames through which these data might be interpreted, our focus is on the mediating roles that patient organization representatives and their organizations play in connecting the lived experiences of patients to the research processes of stem cell researchers and which rationales inform these interactions.

We acknowledge some criticisms of Habermas’ theory regarding its attempt to integrate the relations between social systems and everyday life actions within a single theory, such as those made by Haferkamp (1985). These critiques suggest that Habermas’ theory may be unnecessary, as traditional action theory used in sociology could be sufficient to explain the differences between the everyday world and social systems. Despite this argument, we still contend that Habermas’ work, particularly where it highlights this detachment between everyday actions and social systems, can offer valuable insights to this project. His concept of communicative action provides a useful framework for analysing how researchers and patient organization representatives adapt communicative strategies and influence both the lifeworld and rational systems.

## Supplementary Information

Below is the link to the electronic supplementary material.

Supplementary file1 (DOCX 24 kb)

## Data Availability

The dataset for this study was obtained from human participants, and, given the limited population pool from which participants were recruited in Australia, releasing the raw data would pose a significant risk of identification. As part of the informed consent process, participants were assured that all reasonable measures would be taken to safeguard the confidentiality of their personal information. In accordance with this commitment, the raw data cannot be made publicly available. However, the analysed data (including codes and themes)may be made available upon request (which would be reviewed against the informed consent signed by participants).

## Footnotes

1 We recognize that the use of the term “representative” is disputed (this will be discussed later in this paper). However, in this paper, we use the term “patient organization representatives” when referring to our interviewees, as it is the term they use to self-identify. This also indicates that individuals may have varying roles within these organizations, such as being formally employed due to their professional background or contributing as volunteers, whilst also being patients themselves or carers of individuals with a health condition.
