# Supplementary material for: Exploring Patient Organization Involvement in Stem Cell Research: Insights from the Lifeworld
Source: J Bioeth Inq. 2026 Mar 17;23(3):699–713. doi: 10.1007/s11673-025-10516-6 (PMC13645905; doi:10.1007/s11673-025-10516-6)
Supplement: Supplementary file 1 [file 11673_2025_10516_MOESM1_ESM.docx]

**Examples of questions from interview guide**

These are only some sample questions as interview guides were adapted according to interviewee type/experience/knowledge of stem cell research

**Sample questions (all interviewees)**

What are your views about stem cell research in general and its clinical applications?

What do you think is the value of sharing scientific knowledge related to stem cell research/therapies with a broad range of groups/people?

How do you find out about stem cell research and therapies conducted in Australia? How would you prefer to find out about stem cell research conducted in Australia?

Trade-offs between public and private/commercialised research and healthcare

If stem cell research results in new health products that could assist patients, what are your views on the commercialisation of these products?

Understanding of science as being done for the ‘public good’

What does the idea of science being done ‘for the public good’ mean to you?

**Sample questions (researchers)**

Do you think there is adequate information on source, provenance, etc. for stem cell lines that you use or would consider using?

Do you think that it would be useful for Australia to have a stem cell registry, and if so, what would you like to see integrated within it?

How do other researchers or clinicians find you and learn about your research?

How could communication between different groups (e.g. researchers, manufacturers, clinicians, patients) be improved? What would the benefits be of improving communication between these groups?

How do you think transparency affects collaboration between researchers, manufacturers, and clinicians in stem cell research?

General attitudes toward 'open science' and particularly sharing of data, results, lines, etc. (and broader aspects of open science such as open access, open reviewing, open science tools)

How would you define 'open science'? Do you think it is particularly important in stem cell research and in what ways?

With respect to stem cell research, what parts should be 'open', and what does being 'open' mean in this context?

General attitudes toward commercialisation of outputs including stem cell lines and similar (and trade-offs with openness and data sharing)

To what extent do you think the outputs of stem cell research should be commercialised? Why? What tensions or trade-offs are there between commercialisation and openness (in its

various forms)?

What tensions do you see between sharing information or data from research, whilst also upholding requirements for privacy/confidentiality? How could these tensions be better balanced (resolved)?

Regarding stem cell research and therapies, how do you think information could be made more accessible to various stakeholders?

Generally, what is your sense of Australian public views on stem cell research?

Who is 'missing out' on the potential benefits associated with stem cell research and therapeutics

Do you think that there are particular groups that may be overlooked with respect to stem cell research, in terms of: 1) who is involved in discussions about it, and 2) who may potentially benefit from it?

What issues about stem cell research are not currently being discussed that you think deserve more attention?

**Sample questions (researchers/manufacturers)**

What sorts of products/services related to stem cell research are you (the manufacturer) producing?

What is the purpose of making these products/services and why they are important?

Who are your clients/end-users? (types)

Are there products/services that cannot currently be produced in Australia?

To your knowledge, how many stem cell models have been made from your organisation/company that are available for commercial use and non-commercial use respectively?

Do you think there is adequate information provided to potential users on source, provenance, etc. for stem cell lines that you manufacture?

How do researchers find out about your lines and the associated research?

How could communication between different groups (e.g. researchers, manufacturers, clinicians) be improved?

What would the benefits be of improving communication between these groups?

General attitudes toward 'open science' and sharing of data, results, lines, etc. (and broader aspects of open science such as open access, open reviewing, open science tools)	How would you define 'open science'?

What are benefit of it, what are the obstacles or problems with it?

What role could manufacturers play in making research (more) 'open'?

General attitudes toward commercialisation of outputs including stem cell lines and similar (and trade-offs with openness and data sharing)

Familiarity with stem cell registries: Have you used a registry? (prompt with examples: hCSPreg, SKIP, etc.) What were your motivations?

What are the incentives/barriers to use? Can you please show us how you used it?

What are the key components of a stem cell registry? (e.g., cell line identity,

Would it be useful or duplicative to have an Australian stem cell registry, and why?

What considerations would be important to think about if an Australian stem cell registry were to be established? (e.g. regulatory requirements, implications for collaborations both within Australia and with overseas researchers etc.)

Values underlying a well-functioning registry

Generally, what is your sense of Australian public views on stem cell research?

What are some barriers or challenges with improving how 'trustworthy' stem cell research is perceived to be?

Who is 'missing out' on the potential benefits associated with stem cell research and therapeutics

How do manufacturers currently engage/interact with the public (or particular publics)?

What role (if any) should manufacturers have in engaging with the public?

Understanding of science as being done for the ‘public good’

**Sample questions (researchers/clinicians)**

Do you think there is adequate information on source, provenance, etc. for stem cell lines that you might recommend to a patient?

Do you think that it would be useful to clinicians like you for Australia to have a stem cell registry, and if so, what would you like to see integrated within it?

How could communication between different groups (e.g. researchers, manufacturers, clinicians) about stem cell research and therapies be improved?

How would you define 'open science'? Do you think it is particularly important in stem cell research and in what ways?

With respect to stem cell research, what parts should be 'open', and what does being 'open' mean in this context?

General attitudes toward commercialisation of outputs including stem cell lines and similar (and trade-offs with openness and data sharing)

What tensions are there between commercialisation and openness (in its various forms)?

What tensions do you see between sharing information or data for research, whilst also upholding requirements for privacy/confidentiality? How could these tensions be balanced (resolved)?

Generally, what is your sense of Australian public views on stem cell research?

What are some barriers or challenges with improving how 'trustworthy' stem cell research is perceived to be?

Who is 'missing out' on the potential benefits associated with stem cell research and therapeutics	Do you think that there are particular groups that may/are being overlooked with respect to stem cell research, in terms of: 1) who is involved in discussions about it, and 2) who may potentially benefit from it?

What issues about stem cell research are not currently being discussed that you think deserve more attention?

**Sample questions (patient organisation)**

Generally, to what extent are those you represent interested in stem cell research and its potential applications?

What factors might influence this interest (either positively or negatively), and why?

What factors influence the extent of your community’s trust in (both positive and negative ways), 1) the medical sector, broadly speaking, and 2) stem cell research, in particular?

Is there anything specific about stem cell research as a medical field/application that affects your trust (and the trust of those you represent)?

What are the main issues/problems (medical or otherwise) that your group is trying to address?

To what extent could stem cell research contribute to addressing these main issues/problems?

What sources/outlets of information are most useful for those you represent to learn more about emerging research of clinical relevance?

What makes these sources/outlets reliable, trustworthy and/or accessible?

What sorts of information does your group need to better engage with stem cell research and therapeutics? How do you think this information should be made available/accessible?

How could communication between different groups (e.g. researchers, manufacturers, clinicians) about stem cell research and therapies be improved?

In striving to improve research and develop helpful treatments, what tensions do you see between data privacy and openness?

In what way/s are the patients/groups you represent involved in research that they believe could be beneficial?

In what way/s could this involvement be improved or fostered?

Does the group you represent feel that Australian medical research is aligned with public good? Why or why not?

Perceived trade-offs between commercialiation and making research 'public'

Under what circumstances (if any) could it be beneficial to commercialise the outputs from stem cell research?

What criteria should be used to set priorities for stem cell research and developing therapeutics?

What issues about stem cell research are not currently being discussed that you think are deserve more attention?
